# Mutational Landscape of the Proglucagon-Derived Peptides

**DOI:** 10.3389/fendo.2021.698511

**Published:** 2021-06-17

**Authors:** Peter Lindquist, Jakob S. Madsen, Hans Bräuner-Osborne, Mette M. Rosenkilde, Alexander S. Hauser

**Affiliations:** ^1^ Department of Biomedical Sciences, Faculty of Health and Medical Sciences, University of Copenhagen, Copenhagen, Denmark; ^2^ Department of Drug Design and Pharmacology, Faculty of Health and Medical Sciences, University of Copenhagen, Copenhagen, Denmark

**Keywords:** proglucagon, pharmacogenomics, GLP-1, GLP-2, glucagon, GPCR, mutant, GCG

## Abstract

Strong efforts have been placed on understanding the physiological roles and therapeutic potential of the proglucagon peptide hormones including glucagon, GLP-1 and GLP-2. However, little is known about the extent and magnitude of variability in the amino acid composition of the proglucagon precursor and its mature peptides. Here, we identified 184 unique missense variants in the human proglucagon gene *GCG* obtained from exome and whole-genome sequencing of more than 450,000 individuals across diverse sub-populations. This provides an unprecedented source of population-wide genetic variation data on missense mutations and insights into the evolutionary constraint spectrum of proglucagon-derived peptides. We show that the stereotypical peptides glucagon, GLP-1 and GLP-2 display fewer evolutionary alterations and are more likely to be functionally affected by genetic variation compared to the rest of the gene products. Elucidating the spectrum of genetic variations and estimating the impact of how a peptide variant may influence human physiology and pathophysiology through changes in ligand binding and/or receptor signalling, are vital and serve as the first important step in understanding variability in glucose homeostasis, amino acid metabolism, intestinal epithelial growth, bone strength, appetite regulation, and other key physiological parameters controlled by these hormones.

## Introduction

Blood glucose homeostasis is an essential process and is extensively controlled by a series of peptides derived from the 180-amino acid preproglucagon, encoded by the *GCG* gene. In the early 1980s proglucagon amino acid sequences were first determined from anglerfish isolates, followed by hamster and human cDNAs, which revealed that glucagon and two related glucagon-like peptide (GLP) hormones were derived from a larger preprohormone (1, 2). The identification and understanding of the physiology of proglucagon-derived peptides has paved the way for therapeutic agents for the treatment of type-2-diabetes (T2D), short bowel syndrome, obesity, and acute hypoglycemia in diabetic patients, projected to comprise 700 million people by 2045 (3–6). Moreover, dysregulation of insulin secretion and glucose metabolism can contribute to neurodegenerative Alzheimer’s disease (7).

**Figure 1:**
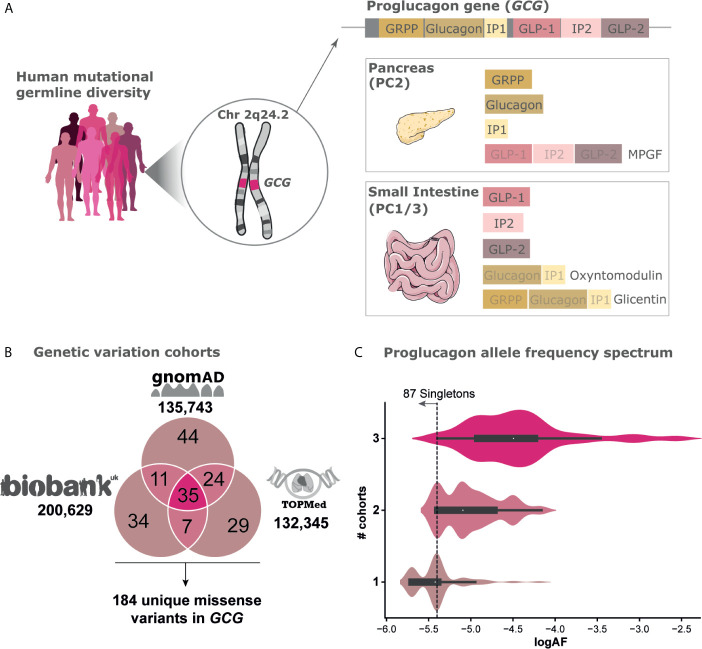
Genetic variation of the Proglucagon peptide hormone gene. **(A)** Human germline genetic variation diversity of the proglucagon gene *GCG* located on chromosome (2q24.2) with expression mainly in the pancreas and the small intestine (see gtexportal.org for expression data). Cleavage enzymes differently cleave the proglucagon precursor into distinct peptides. **(B)** Cross-sectional mutational landscape aggregated from three independent genomic sequencing efforts including gnomAD [122,439 exomes/13,304 genomes, excluding individuals also found in TOPMed (12)], UK Biobank [200,629 exomes (13)], and TOPMed [132,345 genomes (14)] leading to a total set of 184 unique missense variants spanning 117 positions found in a total set of 468,717 unique individuals (**SI2**). **(C)** Allele frequency (AF) spectrum of variants found in one, two, or all three cohorts respectively. The threshold for singletons, i.e., variants only carried by a single individual within a given cohort, are highlighted.

Proglucagon is produced from preproglucagon by cleavage of the 20 amino acid long signal peptide. Tissue-specific enzyme prohormone convertases (PC) 1/3 and PC2 further cleave proglucagon at pairs of dibasic amino acid sequences, except at the GLP-1 NH_2_-terminal site represented by a single Arg residue (8). In pancreatic α-cells, proglucagon is enzymatically processed by PC2, which liberates glucagon, glicentin-related polypeptide (GRPP), major proglucagon fragment (MPGF), and intervening peptide-1 (IP-1) (9). In intestinal enteroendocrine L-cells proglucagon is post-translationally processed by PC1/3 cleaving into glucagon-like peptide-1 (GLP-1 (7-36NH2)), glucagon-like peptide-2 (GLP-2), oxyntomodulin, glicentin, and intervening peptide-2 (IP-2) (8, 10, 11) (**Figure 1A**).

Glucagon was the first proglucagon-derived peptide to be discovered. It was first identified in 1923 and its structure was determined in 1953. In 1959 Roger Unger reported the first development of a glucagon radioimmunoassay (15). Glucagon is thought to have originated first around 1 billion years ago with GLP-1 and GLP-2 created about 300 million years later by exon triplication of ancestral glucagon (16, 17). Such replication events, in which new genes and gene functions can evolve, are vital to the origin and evolution of species (18). The glycogenolytic function of glucagon and its role as a central hormone in blood glucose homeostasis is preserved in all vertebrates - from jawless fish to primates. This is reflected in its considerable sequence conservation, with more than 72% similarity to human for the most known divergent sequence, the sea lamprey (19). GLP-1 and GLP-2 are likewise well conserved among vertebrates but show slightly larger sequence variation between species compared to glucagon. The other peptides GRPP, IP-1, and IP2 vary more in amino acid lengths with less conservation between species, suggesting that there is less constraint to preserve sequence information (18).

Mutations in the human genome may cause dysregulation of physiological functions leading to diseases and can even change drug efficacy and safety (20, 21). Large-scale sequencing efforts have led to the identification of a rapidly growing number of such single nucleotide polymorphisms (SNPs), with the Single Nucleotide Database dbSNP listing over 700 million unique variants found in human genomes (22). Each individual is carrying about 10,000-15,000 missense variations that alter the amino acid compositions of the resulting proteins (14, 23). Only a fraction of these has been characterized and few associated with disease. The proglucagon peptide hormones signal through class B1 G protein-coupled receptors (GPCRs). GPCRs mediate the therapeutic effect of approximately 34% of drugs on the market (24). Recent studies have shown extensive variability in GPCRs, and mutations within the coding region can lead to several monogenic diseases or to altered drug responses (25, 26). Therefore, missense variants in *GCG*, coding for the hormones may play a role in metabolic diseases or affect the treatment of these.

Class B1 GPCRs mediate signal transduction of the proglucagon hormones GLP-1, GLP-2, glucagon, and oxyntomodulin. It comprises 15 receptors in humans including the GLP-1 receptor (GLP-1R), the GLP-2 receptor (GLP-2R), and the glucagon receptor (GCGR), each of which is stimulated mainly by their respective hormones GLP-1, GLP-2, and glucagon (8, 15, 27, 28). Oxyntomodulin however is capable of signaling through both the GLP-1R and GCGR (29), and glucagon has a functionally important although relatively low affinity towards the GLP-1R (30). The class B1 receptors are characterized by a large N-terminus composed of approximately 100-160 amino acids; a region that serves as the initial contact area with the C-terminus of the endogenous peptides which thereafter position their N-terminus into the transmembrane receptor binding pocket in the 7-transmembrane domain. Ligand binding leads to receptor conformational changes and activation of respective downstream pathways (31–33).

Access to large datasets of human genome sequences comprises a valuable resource for understanding how genetic variation can be associated with disease etiology. Population genetics can reveal sites under active selection and mutational information can highlight structure-function relationships important for receptor recognition, binding, and activation (34, 35). Previous studies have shown structure-function relationships, using systematic alanine substitutions for glucagon, GLP-1, and GLP-2, and characterized the role of individual amino acid positions (36–42). While previous studies have investigated genetic variability in the proglucagon gene across species (18), no studies have examined the prevalence and spectrum of human genetic variation in the proglucagon gene and its derived peptides within the human population. The characterization of genetic variations in terms of their possible impact on activation, selectivity, signaling and beyond, is vital for disease discovery and diagnostics (43). Here, we combine diverse large-scale genetic variant datasets to extensively chart the mutational landscape and to provide insights into the spectrum of genetic variation of the proglucagon gene. This includes the TOPMed database (132,345 genomes), the Genome Aggregation Database (gnomAD; 122,439 exomes and 13,304 genomes that do not overlap with TOPMed), and the UK Biobank (200,639 exomes) (12–14) totaling more than 450,000 individuals. We furthermore include evolutionary conservation metrics, incorporate literature annotations from structure-function studies, and discuss possibly deleterious consequences for variants across the different proglucagon regions providing a perspective for future studies on genetic and pharmacological investigations.

## Results

### High Diversity of Human Missense Variations in the Proglucagon Gene

Although the physiological importance of the proglucagon-derived peptides is well described, genetic variations in the *GCG* gene have not been directly studied. We set out to map genetic variations in the *GCG* gene and investigate the prevalence of mutations across the peptide hormones and their potential impact on receptor interaction (**Figure 1A**). For this analysis, we integrated data from three independent whole exome sequencing (WES) and whole genome cohort studies including aggregated data from both gnomAD and TOPMed and individual data from UK Biobank (12–14). We have focused on missense variants as these are more likely to impact protein structure-function and are diverse yet frequent in the human population. Loss-of-function mutations are often deleterious and hence retained at very low frequencies in the human population (12). With this, we have charted the mutational landscape of the proglucagon precursor in a human population spanning more than 450,000 individuals.

The data from gnomAD consists of aggregated genetic sequence information derived from 122,439 exomes and 13,304 genomes from unrelated individuals across six global and eight sub-continental ancestries (12). Here, we identified 114 missense variants in the *GCG* gene with a global observed over expected (O/E) ratio of 1.04 (confidence intervals 0.89-1.22) (**Figure 1B**). The O/E ratio is an evolutionary constraint score that measures how tolerant a gene is to missense variations by comparing the number of observed variants with the variant count predicted by a depth corrected model of mutational probability (12). An O/E ratio of 1 suggests that missense variations in *GCG* are not under strong selection, which is in line with mouse studies, where *GCG* knock-out offspring experienced no gross abnormalities (44).

The TOPMed database comprises 80 different studies with a cohort of approximately 155,000 ethnically and ancestrally diverse participants. TOPMed contains 132,345 genome sequences not overlapping with gnomAD and these yielded 95 *GCG* missense variants (14).

The UK Biobank contains 200,639 exomes from individual UK participants (13) from which we identified 87 missense variants within the *GCG* gene. Most individuals within the UK Biobank are homozygous for the reference allele across all variants, with only 1550 heterozygote individual variant carriers. Of these, four individuals were heterozygous carriers for two or more variants at distinct sites and only one individual was homozygous for a non-reference allele (I158V^GLP-2,13^). Altogether, the combined investigation across 468,717 individuals identified 184 unique missense variants found across 117 amino acid positions (65%) of the proglucagon gene (**Figure 1B** and **SI1**). Of note, these make up only a subset of the 1229 theoretically possible missense variants in *GCG* resulting in 1072 unique amino acid substitutions which we found by enumerating amino acid substitutions resulting from every possible SNP in the gene (45). The allele frequency spectrum of genetic variants either found in one, two, or all three of the analyzed cohorts is highly diverse. As expected, the 35 genetic *GCG* variants found in all three cohorts display a higher mean allele frequency (mean AF: 3.2x10^-5^) (**Figure 1C**) compared to variants found in two (mean AF: 8.1x10^-6^) or only in individual datasets (mean AF: 4.0x10^-6^). Nearly half (87) of all genetic variants identified are singletons, i.e. variants only carried by a single individual within a given cohort. The singletons have an estimated allele frequency of roughly 1 in 260,000 to 1 in 400,000 (UK Biobank) corresponding to a log allele frequency (log_10_AF) of -5.4 or -5.7, respectively.

### Glucagon, GLP-1 and GLP-2 Are More Conserved and More Likely to Be Functionally Impacted by Genetic Variation

**Figure 2:**
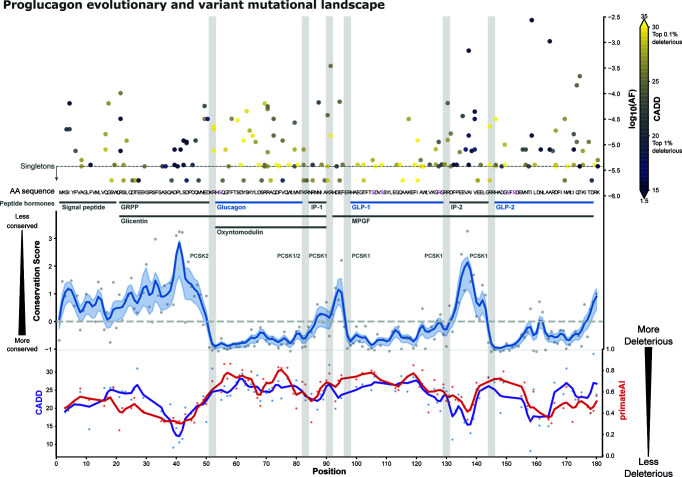
Proglucagon mutational landscape. Functionally described peptide hormones are highlighted (*blue)*, which are more conserved in an evolutionary trace analysis employing Rate4Site scores [blue line, gaussian CI: 50% (46, 47)], than other parts of the precursor and peptides such as IP1/IP2, GRPP, and the signal peptide (most conserved position has a score of –1). All 184 missense variants are displayed along their predicted CADD scores (*color-grading*) (48) and their corresponding max allele frequencies (*top-right y-axis*). Peptide cleavage motifs are highlighted as grey bars alongside their known enzymes. Post-translational modification sites are highlighted (*pink*) in the amino acid sequence. Predicted *CADD* (*purple*) and *primateAI* (*red*) scores are presented (*bottom curves*) (48, 49), indicating higher mean predicted deleteriousness in glucagon and GLP-1. See **SF1** for an interactive version.

Given the aggregated variant information, we analyzed the genetic variation with respect to their genetic location by mapping all missense variants across the 180 amino acid preproglucagon sequence (see **Figure 2** and **SF1, SI1**). Most of the missense variants are located in sites encoding for peptides (165 out of 184), with the GLP-2 peptide exhibiting most unique variants (35) and IP-1 the fewest (5), which are also the longest and shortest peptides respectively. Taking the peptide’s length into account, IP-2 exhibits the highest density of variation (80% of positions) and GLP-2 the lowest density (61%).

The allele frequency spectrum represents both low-frequent variants such as singletons only found in single samples and variants with higher frequency such as I158V^GLP-2,13^ found in 1 in 492 individuals (mean AF across cohorts: 0.27%). Furthermore, the GLP-1 variant K117N^GLP-1,26^ and the glucagon variant R70H^Glucagon,18^ are among the most frequent, respectively occurring 1 in 33,282 and 1 in 23,951 individuals.

To better understand the functional impact of these mutations, we employed combined annotation-dependent depletion (CADD) scores to predict likely deleterious genetic variants (**Figure 2**) (48). CADD is based on a logistic regression model using more than 60 different annotations including conservation, selection, and functional features. CADD scores are scaled such that a score of 10 corresponds to the variant being among the top 10% most deleterious among all, ~9 billion, possible genetic variants, while a score of 20 reflects the top 1% etc. The mean CADD score for *GCG* variants was 21.9 where 15 is the median score when considering only non-synonymous variants, with individual sites displaying considerable variation. The genetic variant A115G^GLP-1,24^ exhibited the highest CADD score of 28.9 among the GLP-1 variants, which is a singleton found in the UK Biobank. Among the glucagon variants, Y62C^Glucagon,10^ displayed the highest CADD score of 29.3 (AF: 1.46x10^-5^). Based on CADD, A115G^GLP-1,24^ and Y62C^Glucagon,10^ are among the most putatively deleterious variations. When looking at those alleles with a CADD score > 20, we identify 375 heterozygous individuals from the UK Biobank carrying potentially deleterious alleles.

**Figure 3:**
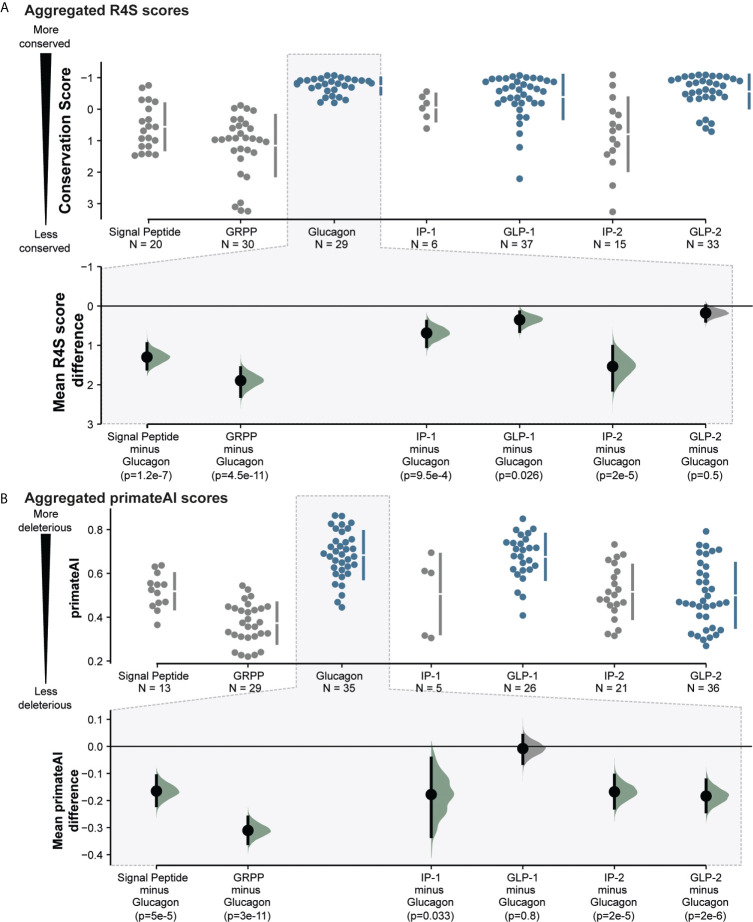
Proglucagon peptide hormones are more conserved and more likely to be severely impacted by missense mutations observed in the human population. **(A)**. Aggregated peptide mean evolutionary conservation scores from evolutionary trace Rate4Site scores, showing significant conservation of glucagon, GLP-1 and GLP-2 peptides (*highlighted in blue*). **(B)** Aggregated primeateAI scores across peptides, indicating higher predicted detrimental effects on glucagon, GLP-1 and GLP-2 peptide hormones. Mean difference analysis (*bottom*) from *dabest* (50) comparing other peptides to glucagon. P-values were calculated by a nonparametric Mann–Whitney test and distributions have been highlighted in green if below 0.05. See **SI2** for more detailed information.

To assess the evolutionary conservation of aminoacid sites in specific regions of *GCG*, we employed an evolutionary conservation score to detect sites subject to purifying selection. Based on a multiple-sequence alignment (MSA) of 222 high confidence orthologues from 164 vertebrates, we used Rate4Site (R4S) to estimate the relative evolutionary rate for each position (46). With lower conservation scores, residues within GLP-1, GLP-2, and glucagon appeared more conserved than the other proglucagon-derived peptides (**Figure 2** and **SF1, SI1**). We aggregated R4S scores for each peptide to investigate potential differences between the peptides. This analysis shows that glucagon is most conserved, i.e. has the lowest mean evolutionary conservation score, (mean R4S: -0.70) followed by GLP-2 (-0.54) and GLP-1 (-0.24) (**Figure 3A** and **SI2**). With glucagon as the most conserved peptide reference, we compared mean R4S scores across peptides, which revealed that glucagon is significantly more conserved compared to the functionally lesser known proglucagon-derived peptides GRPP, IP-1, IP-2, and the signal peptide (Mann–Whitney test; SP: p ≤ 1.2x10^-7^; GRPP: p ≤ 4.5x10^-11^; IP-1: p ≤ 9.5x10^-4^; IP-2: p ≤ 2.0x10^-5^). This suggests a higher degree of purifying selection for glucagon, GLP-1, and GLP-2 with an evolutionary constraint to preserve their function (51). This approach has previously been employed to identify new peptide hormones in known or putative precursor proteins highlighting evolutionary conservation as an important indicator for functional importance (52).

In addition to the CADD score, we extended the analysis with another functional predictor, PrimateAI. Here, a deep neural network predicts variant deleteriousness based on learned local secondary structure prediction as well as primate and vertebrate orthologue sequence alignments (45). CADD scores and PrimateAI score correlate and illustrate a similar pattern in the variants’ predicted deleteriousness (Pearson’s correlation: 0.65; p ≤ 2.2x10^-23^). We observe higher mean PrimateAI scores, and hence higher predicted deleteriousness, for glucagon (0.683), and GLP-1 (0.676), but similar scores for GLP-2 (0.5) and IP1/2 (0.51) (**Figure 3B** and **Supplementary Figure 1**, **SI2**). With the same approach as for the R4S scores, we compared the mean PrimateAI scores of glucagon across the panel of peptides, which shows a similar pattern highlighting glucagon sequence variations to be more deleterious than other proglucagon-derived peptides (Mann–Whitney test; SP: p ≤ 5.0x10^-5^; GRPP: p ≤ 3.0x10^-11^; IP-1: p ≤ 0.033; IP-2: p ≤ 2.0x10^-5^). This indicates that the evolutionary more conserved peptides corresponding to GLP-1, GLP-2 and glucagon also displayed higher mean predicted deleteriousness (Pearson’s correlation primateAI vs. R4S: 0.55; p ≤ 3.0x10^-16^).

### Energy Calculations Point Towards Glucagon and GLP-1 Mutations Likely to Impact Receptor-Ligand Interactions

Protein-protein interactions (PPIs) are essential for physiological functions such as signaling transduction through GPCRs. Thermodynamic information can describe the strength of PPIs or binding free energy ΔG. Mutation-induced binding affinity changes (i.e., ΔΔG in kcal/mol) can be estimated through physical energies and statistical potentials by calculating the difference of binding affinity between mutant and wildtype receptor-ligand complexes (see **methods**) (53). Based on this, we characterized the putative impact of both GLP-1 and glucagon missense variants by calculating their folding complex energies given the availability of high-resolution structures for both GLP-1 in complex with the glucagon-like peptide-1 receptor (GLP1R) (PDBid: 6X18) (54) and glucagon in complex with the glucagon receptor (GCGR) (PDBid: 6LMK) (55).

**Figure 4:**
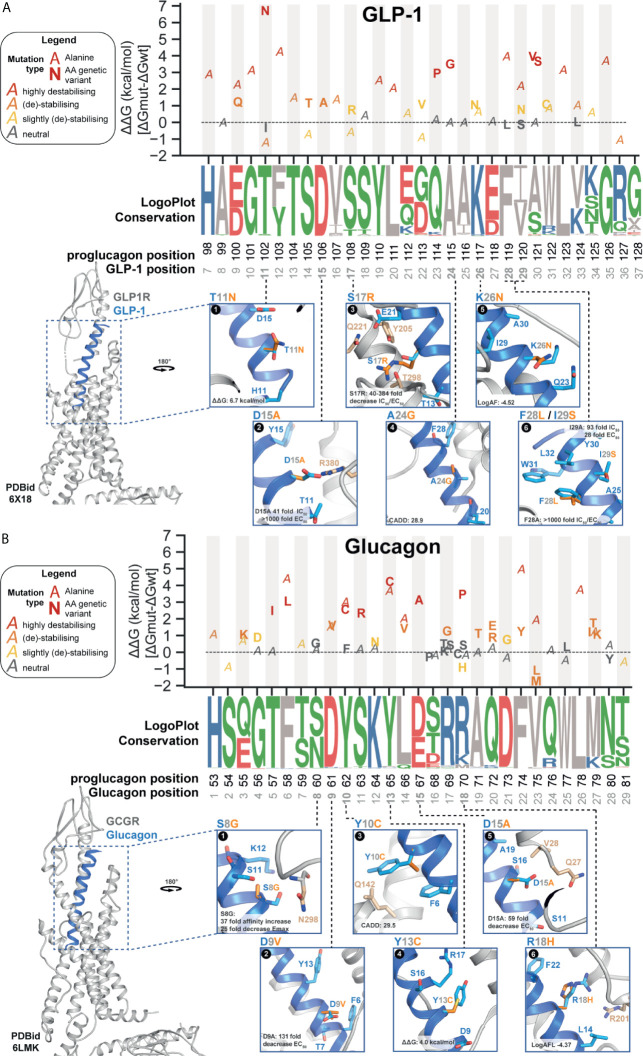
Proglucagon peptide hormone receptor structure and ligand-interaction with wild type (WT) or mutant genetic variant. **(A, B)** Predicted binding affinity changes (*top panel*) ΔΔG (kcal/mol) for genetic variations (glucagon: n=17; GLP-1: n=34) and alanine substitutions based on ΔΔG energy calculations on 10 independent runs from refined structure models (GLP-1 PDBid: 6X18; glucagon PDBid: 6LMK) (56). All genetic variants (marker: *amino acid variant in bold*) found in the combined set of genetic variant data (**Figure 1A**) and alanine substitutions for all positions in the respective receptor complexes (marker: *A*). Variants are colored based on different levels of energy, red (highly destabilizing), orange (destabilizing), yellow (slightly destabilizing), and grey (neutral). Evolutionary conservation on logo-plots (57) based on a multiple-sequence alignment (from Ensembl Compara (58),) of 222 high-confidence orthologues from 164 vertebrates (including in-paralogs). Each letter’s height represents the frequency within the aligned sequences ordered from the most conserved on the top of the letter stack. Polar contacts to the selected WT peptide position displayed in stick format. Peptide residues (*light blue*), genetic variant (*orange*), receptor residues (*beige*). Max fold change in IC_50_ and EC_50_ for the genetic variants presented with CADD score describing the genetic variant’s predicted deleteriousness. **(A)** Representation of glucagon-like peptide-1 receptor (GLP-1R) structure (*grey*) in complex with GLP-1^7-36-NH2^ (*blue*) (PDBid: 6X18). **(B)** Representation of the glucagon receptor (GCGR) structure (*grey*) in complex with glucagon^1-29^ (*blue*) (PDBid: 6LMK). See **SI3** and **SI4** for more information.

We performed a systematic *in silico* alanine substitution scan of all GLP-1 and glucagon residues in addition to all observed missense variations, estimating the impact on binding affinity (**Figure 4** and **SI3**). The replacement with alanine was used to investigate if specific positions are crucial for mediating ligand-receptor interaction or specific polymorphisms that result in destabilizing interactions. This approach rendered several GLP-1 and glucagon variants likely to cause an unfavorable increase in binding free energy, potentially impairing endogenous receptor-ligand interactions. This may further influence glucagon control suggested to be defected in some patients with T2D (59), as well as GLP-1 action and secretion contributing to insufficient insulin secretion (8, 60).

A positive ΔΔG value indicates a less energetically favorable ligand-receptor interaction, hence causing destabilization of the complex. Vice versa, a negative ΔΔG value suggests that the mutation stabilizes the receptor-ligand complex. We classified variants into four categories based on the calculated energy change kcal/mol: highly destabilizing (>+1.84 kcal/mol), destabilizing (+0.92 to +1.84 kcal/mol), slightly destabilizing (+0.46 to +0.92 kcal/mol), and neutral (-0.46 to –0.46 kcal/mol) (61). Overall, consistent with the structural conservation between glucagon and GLP-1 (and similar class B1 peptide hormones), we found overlap in terms of amino acid positions of impact for peptide stability. In total, we found seven variants (21%) classified as highly destabilizing for glucagon and five for GLP-1 (~30%) with GLP-1 variants being on average slightly more destabilizing than for glucagon (1.63 vs. 1.00 mean kcal/mol) (**Figure 4** and **SI3**).

For the GLP-1 SNPs, the thermodynamically most destabilizing variants (ΔΔG >1.84 kcal/mol) are T102N^GLP-1,11^, Q114P^GLP-1,23^, A115G^GLP-1,24^, and A121S/V^GLP-1,30^. The singleton variant T102N^GLP-1,11^ is the GLP-1 variant with the highest calculated ΔΔG with an energy change of +6.74 kcal/mol - mostly resulting from Van der Waals clashes (**Figure 4A-1**). While there is no *in vitro* characterization data available for T102N^GLP-1,11^, data from an alanine scan showed a 13-fold change in binding affinity but only a 2-fold change in potency for T102A^GLP-1,11^ (**SI4** for literature annotations of peptide mutant effects) (37). The ΔΔG energy for the alanine substitution resulted in a -1.19 kcal/mol change in binding free energy, classifying this substitution as energetically favorable and stabilizing (61), hence supporting the *in vitro* data for T102A^GLP-1,11^ and suggesting an important contribution of the amide group. Thr11^GLP-1^ contributes to stabilizing the GLP-1 N-capped conformation of the GLP-1 N-terminus, which was proposed to be a structurally important element for receptor activation (62, 63).

For the SNPs in glucagon, the highly destabilizing hot spots in glucagon were found to be T57I^Glucagon,5^, F58L^Glucagon,6^, Y62C^Glucagon,10^, S63R^Glucagon,11^, Y65C^Glucagon,13^, D67A^Glucagon,15^ and R70P^Glucagon,18^ all with a ΔΔG > 1.84 kcal/mol. A calculated ΔΔG of 4.18 kcal/mol makes Y65C^Glucagon,13^ (AF: 5.0x10^-6^) the variant with the least energetically favorable receptor-ligand interaction among all glucagon variants, suggesting structural importance of the benzene ring, which is disrupted by thiol-containing cysteine substitution (**Figure 4B-4**). Energy calculations for F74Y^Glucagon,22^ (AF: 2.6x10^-6^) indicate destabilizing energy contributions (ΔΔG of 1.19 kcal/mol). *In vitro* alanine substitution at this position resulted in a 622-fold potency decrease (38), which is supported by the free binding energy calculations rendering the F74A^Glucagon,22^ variant as the most destabilizing alanine substitution (ΔΔG of 4.98 kcal/mol) (**SI3**). This indicates that this position is important for receptor activation and that even minor alteration such as the added hydroxyl group in F74Y^Glucagon,22^ might impact receptor signaling.

We further selected individual variant outliers with high allele frequency, CADD/primeateAI scores, ΔΔG, and variants pharmacological characterized by *in vitro* examination to further illustrate GLP-1 and glucagon interactions in complex with their respective receptors (**Figure 4A, B** and **SI3/4**).

The singleton variant D106A^GLP-1,15^ was investigated *in vitro* by alanine scanning in structure-activity studies in the 1990s and found to decrease the binding affinity >40-fold and to decrease cAMP activity > 1000-fold (**Figure 4A-2** and **SI4**) (37). It has been suggested that the acidic D106^GLP-1,15^ interacts with the basic GLP-1R residue R380^7x34^ possibly through electrostatic attraction crucial for ligand-induced receptor activation, carried out by the positively (Arg) and negatively (Asp) charged side chains. The aliphatic amino acid alanine disrupts this interaction, also indicated by our free binding energy calculations classifying the Ala substitution as destabilizing (ΔΔG > 0.92 kcal/mol) (64). Another study examined the GLP-1 variant S108R^GLP-1,17^ (AF: 2.6x10^-6^) (36). The mutated ligand resulted in a 104-fold decrease in binding affinity and a 112-fold ED_50_ decrease in insulinotropic activity (**Figure 4A-3**) (36). The variant F119L^GLP-1,28^ and I120S^GLP-1,29^ have been described as being part of the hydrophobic face interacting with the GLP-1 extracellular domain (ECD) (**Figure 4A-6**) (65). *In vitro* alanine scan of these positions resulted in a 1300-fold affinity and 1040-fold potency decrease for F119L^GLP-1,28^ as well as a 92-fold affinity and 28-fold potency decrease for I120S^GLP-1,29^ (37). This is supported by ΔΔG calculations indicating the alanine substitutions as highly destabilizing (F119L^GLP-1,28^: ΔΔG of 3.9 kcal/mol; I120S^GLP-1,29^: ΔΔG of 2.1 kcal/mol; **Figure 4A-6** and **SI3**).

For glucagon, the S60G^Glucagon,8^ (AF: 1.07x10^-5^) is the only variant, which has been specifically investigated (40). It demonstrated a 31-fold decrease in binding affinity and a 25-fold decrease in adenylyl cyclase activation (40). This highlights a potentially important polar ligand-receptor interaction between glucagon and GCGR residue N298^ECL2^ not formed in the presence of glycine (**Figure 4B-1**). Another alanine scan identified the N-terminal region as highly intolerant to alanine substitutions including the variant D67A^Glucagon,15^ (AF: 1.34x10^-6^), which was found to lead to a 59-fold potency loss (38). This indicates that an essential polar interaction between the charged D67^Glucagon,15^ and the GCGR residues Q27^ECD^ and V28^ECD^ is diminished by the structurally distinct alanine (**Figure 4B-5**). These findings are supported by previous results, suggesting position 15 as essential for receptor recognition (39). Replacement of Asp in glucagon at position 9 has demonstrated to impair stimulation of adenylyl cyclase (42). The variant D61V^Glucagon,9^ (AF: 1.34x10^-6^). An *in vitro* alanine scan showed that D61A^Glucagon,9^ exerted the second greatest loss in potency (131-fold) (**Figure 4B-2**) (38). Free binding energy calculations (ΔΔG >1.55 kcal/mol) of D61A^Glucagon,9^ and D61V^Glucagon,9^ indicated a destabilizing energy contribution (**Figure 4B**). All together supporting that Asp at glucagon position 9 is crucial for receptor binding (42).

## Discussion

While there are numerous studies investigating genetic variants at the protein family level for GPCRs (25, 26), regulators of G protein signaling (66), G proteins (67, 68), and olfactory receptors (69), very little emphasis has been placed on the possible impact of genetic variants of the genes of peptide hormones. This is remarkable given that more than two-thirds of human peptide hormones are targeting GPCRs, with more than 200 peptide ligands originating from 130 different precursor genes (52). While one study investigated the missense variants in six orexigenic neuropeptides important for appetite and energy homeostasis demonstrating how to utilize genome sequence datasets to map SNPs potentially changing receptor signaling (70), the extent to which genetic variants impact receptor-ligand interactions still remains to be elucidated.

Here, we focused on the *GCG* gene and its derived peptide hormones, which are important in various (patho)physiological processes related to glucose metabolism and are associated with diabetes and other disorders. Previously, 29 variants have been identified in the *GCG* gene among 865 Europeans with I158V^GLP-2,13^ as the single missense variant, but no significant association could be found for carbohydrate metabolism in a larger genotyping study (71). We utilized three independent whole-genome and exome sequencing datasets from the gnomAD database, TOPMed, and the UK Biobank to map the mutational landscape of missense variants in the *GCG* gene and to assess their potential effects on receptor signaling. This resulted in 184 unique missense variants from 117 positions in the *GCG* gene identified in a human population of 468,717 individuals.

By integrating various metrics such as allele frequencies, predicted deleteriousness, and evolutionary conservation, we identified clear differences between the functionally well described peptides glucagon, GLP-1, and GLP-2, compared to the other peptide products, GRPP, IP-1, and IP-2. Generally, the established peptide hormones, which exert essential biological functions, exhibit a higher evolutionary conservation and predicted deleteriousness compared to a much lower sequence conservation in the remaining peptides suggesting fewer biological constraint factors (18, 72). This finding is in line with the notion that structurally essential proteins are likely to be more conserved and evolve at a slower rate (51). The N-terminal part of these hormones are important for the receptor activation after initial contact between the α-helical part and the receptor N-terminal, as illustrated by the N-terminally truncated Exendin-(9-39)-amide which not only has no agonist activity but actually is a potent GLP-1 antagonist (73–75). Consistent with this, the non-helical confirmation close to the N-terminal of GLP-1 correlates with greater agonist potency (76) and the residue His1 is conserved in GLP-1, GLP-2, glucagon, and most members of the glucagon-related peptides superfamily (73, 74). This residue’s importance was highlighted by three independent alanine scans showing that alanine substitution of His1 in all three peptides hormones resulted in disruption of ligand binding (37, 38, 41). No genetic variants have been found for His1 in GLP-1 and glucagon, and only a single individual was identified with a mutated His1 in GLP-2, highlighting that species conservation as well as population conservation can be indicative readouts of functionally important positions.

Human genetic variants are present in 17 out of the 30 GLP-1 residues. Our energy calculations indicated that the variants T102N^GLP-1,11,^Q114P^GLP-1,23^, A115G^GLP-1,24,^and A121S/V^GLP-1,30^ contribute strongly to destabilization of the ligand-receptor complex. Previous *in vitro* alanine scan of GLP-1 indicated that position D15, F28, and I29 were particularly vulnerable to alanine substitution, resulting in 40-, 92-, and 1300-fold decreases in agonist affinity. These positions correspond to the genetic variants D106A^GLP-1,15^, F119L^GLP-1,28^ and I120S^GLP-1,29^ (37). Our calculations of free binding energy classified these alanine substitutions from destabilizing to highly destabilizing. The variant D106A^GLP-1,15^, is thought to be involved in electrostatic interactions with the GLP-1R residue R380^7x34^, possibly explaining the destabilization upon alanine substitution (64). The residues F119^GLP-1^ and I120^GLP-1^ have shown to be a part of the hydrophobic interface of GLP-1 that interacts with the ECD of the GLP-1R (65). However, based on binding energy calculation for F119L^GLP-1,28^ and I120S^GLP-1,29^, these variants did appear thermodynamically unfavorable.

Out of 29 positions, 23 amino acids in glucagon were found to display genetic variants. Energy calculations revealed the variants T57I^Glucagon,5^, F58L^Glucagon,6^, Y62C^Glucagon,10^, S63R^Glucagon,11^, Y65C^Glucagon,13^, D67A^Glucagon,15^ and R70P^Glucagon,18^ to be highly destabilizing for the glucagon ligand-receptor complex. *In vitro* alanine scan of glucagon demonstrated >59-fold decrease in potency for the residues D9, Y10, D15, F22, and V23 (38). This correlates with our free energy calculation classifying the alanine substitutions in the respective positions from destabilizing to highly destabilizing. The variant D67A^Glucagon,15^ has been reported to be fundamental for receptor recognition and involved in important hydrogen bond interaction with GCGR residue M29^ECD^ in agreement with the impaired ligand potency (39, 77). The F22 alanine substitution demonstrated the highest predicted affinity loss (ΔΔG: 4.98 kcal/mol), which correlates with the greatest *in vitro* potency decrease (622-fold). On the other hand, free binding energy calculations showed that the S60G^Glucagon,8^ variant would be tolerated, whereas *in vitro* investigations indicated more than 25-fold decrease in affinity and potency (40). This demonstrates that computational models have their limits in accurately predicting variant effects but are rather useful tools for the assortment of the most impactful ones.

Given the sheer number of genetic variants, in-depth *in vitro* characterizations are likely unfeasible. Rational selection of variants by employing computational models utilizing evolutionary conservation, machine-learning based predictions of deleteriousness, and binding free-energies may guide the selection of a more manageable set of variants to be tested *in vitro*. Obvious criteria for a selection would be a low R4S scores, high CADD/primateAI scores, high ΔΔG energies, positions with impacted efficacy and/or potency from alanine scanning experiments, residues located in the receptor-binding N-terminal end, and variants with high allele frequencies. While we have employed some of the best benchmarked computational models to estimate variant deleteriousness, more than 40 other variant effect predictors have been developed in recent years (78). In addition, more computationally expensive methods such as free energy perturbations and all-atom molecular dynamics simulations could provide a higher-resolution understanding of variant effects (79). However, this also demands more extensive atomic-resolution data, especially for GLP-1 in complex with GCGR as well as structural data for oxyntomodulin.

Alternatively, various cell-based methods can provide a more comprehensive understanding of variant effects. In recent years, the complexity of the GPCR signaling landscape has been become more apparent. Missense mutations found in the proglucagon-derived peptides may not just impact receptor affinity but may show altered expression and secretion into the extracellular domain, altered selectivity between receptors, changed kinetics or internalization rates, switch modality or shift G protein signaling selectivity. Hence, it is important to characterize all selected variants in the right cellular and experimental context to estimate the direct effects on each of the GPCR signaling dimensions (80). For instance, second messenger assays have been widely employed by taking advantage of the strength and signal amplification downstream of the receptor-G protein coupling. Other experimental setups can probe the direct interaction with G protein and arrestins such as by employing bioluminescence resonance energy transfer (BRET)-based biosensors (81, 82) or investigate cumulative cellular responses in real time label-free receptor assays (83). Finally, relevant transgenic animal models and retrospective biobank or cohort studies could be employed to establish a link between patient genotypes with clinical phenotypes.

Surprisingly, no disease-associated mutations, such as from genome-wide association studies (GWAS), have been identified within the coding region of *GCG*. However, a mutation in the dipeptidyl-peptidase 4 (DPP-4), which cleaves and inactivates GLP-1 and GLP-2, has been shown to negatively affect glucose-stimulated GLP-1 levels, insulin secretion, and glucose tolerance (84). Rare genetic variants in the incretin-related genes have been associated with T2D (85), and polymorphisms in the transcription factor 7-like 2 have shown to impair GLP-1-induced insulin secretion (86). This indicates that a single genetic alteration may impact this complex and delicate system rendering someone more or less susceptible to disease etiology. It also underlines that associations are difficult to identify given confounding factors such as age, gender, life-style factors, BMI, disease heterogeneity, and the impact of environmental exposures. Moreover, disease associated variants are less likely to occur in lowly and sparsely expressed proteins such as *GCG* (87). In addition, most of the *GCG* missense variants are very rare, not on commonly used genotype arrays, and hence below GWAS detection-threshold (88). Pooling variants with similar predicted or tested effects may increase the statistical power for putative association with disease (89).

As more and more sequencing data will become available, it is apparent that additional variants for *GCG* will be discovered. Although *GCG* has no reported *de novo* mutations from father-mother-child trio studies indicating a slow mutation rate (90), it has been estimated that at the current gnomAD sample size, the number of observable missense variants from the current human population is still far from saturation (12). Since selection reduces the number of variants in the population, it is expected that we observe significantly fewer variants in the coding region of *GCG* than theoretically possible (1075). Although we focused on missense variants, which are relatively frequent in the population, yet more likely to impact structure and function, other mutations such as mutations in the promoter region, in introns, and synonymous mutations, might also impact the proglucagon-derived peptides through altered transcription efficacy or alternative splicing patterns. This has been the case for carriers of the rs4664447 variant, predicted to disrupt a *GCG* exonic splicing enhancer, who exhibited decreased fasting and stimulated levels of insulin, glucagon and GLP-1 (71). However, the potential impact of such mutations is much more challenging to predict computationally or to determine *in vitro*.

Conservation based methods are commonly used for protein structure prediction and design (91). In this study, our conservation analysis makes use of a set of genomes of adequate sequencing quality including some, such as teleosts, in which *GCG* have evolved different biological activities. This divergent evolution may have affected our conservation scoring, but as more high-quality vertebrate genomes become available, for example through The Vertebrate Genomes Project (92), we may see an increase in power and utility of conservation-based approaches to elucidate mutational and functional constraints.

Based on the gnomAD data, *GCG* displays a high observed/expected score with roughly as many observed missense variants as expected based on a mutational background model (12). This specifies that *GCG* is not under strong selection against missense variants in the human population. However, this model does not take individual allele frequencies or zygosity into account, which seems to be particularly low for *GCG* given less than a handful of homozygous *GCG* missense variant carriers among >450,000 individuals (as a reference, the median number of total homozygous missense variant carriers is 256 among all GPCRs in gnomAD). This may indicate that individual heterozygous variants can be alleviated by other regulatory mechanisms, whereas homozygous carriers are under higher intolerance. On the other hand, homozygous glucagon-GFP knock-in mice lacking all proglucagon derived-peptides are normoglycemic, display improved glucose tolerance and no gross abnormalities (44, 93). Together, this suggests that individual missense variants are likely not disruptive of physiological conditions associated with *GCG*, but rather have the potential to contribute to an altered glucose metabolism and a predisposition to develop disease depending on the affected hormone. For instance, the stop-gained Trp169Ter mutation in GLP-2 has not been shown to significantly associate with carbohydrate metabolism traits suggesting that a single allele is sufficient for adequate GLP-2 levels (71). Besides, other means of regulation such as adapted expression levels, genomic background, buffering mutations or allele-specific expression might offset the effects of deleterious mutations (94, 95). Moreover, the gut microbiota is thought to modulate energy metabolism and to secrete GLP-1 inducing factors that improve glucose homeostasis (96). Given the low number of carriers for the majority of the proglucagon missense variants, it is likely that much larger cohorts will be needed to delineate deleterious from benign mutations.

The discovery and characterization of proglucagon-derived peptides have produced therapeutics as the GLP-2 analog (teduglutide) for short bowel syndrome, multiple GLP-1 analogs (e.g. dulaglutide, liraglutide and semaglutide), and the novel glucagon analog (dasiglucagon) essential for controlling metabolism and blood glucose levels in the treatment of type-2-diabetic patients (3, 4, 6, 97). Understanding how genetic variation can affect a hormone’s endogenous response provides valuable information for future drug discovery, diagnostics of diseases, and ultimately personalized medicine with tailored drug regimens. Despite the clinical importance of proglucagon-derived peptide analogs, the molecular interaction between ligand and receptors is still not fully understood. Future *in vitro* studies may utilize the mutational landscape of proglucagon-derived peptides as the first steps to translate information about genetic variation into the stratification of sub-populations and actionable drug discovery investigations. Furthermore, investigating the consequence of genetic variation in one proglucagon peptide can facilitate our understanding of others - such as consequences within the glucagon sequence may directly inform us about oxyntomodulin and glicentin functions.

In conclusion, we identified 184 unique missense variants in the human proglucagon gene obtained from >450,000 individuals. The most detrimental genetic variants are suggested to be located in the sequence of the highly conserved GLP-1 and glucagon hormones as suggested by evolutionary metrics, deleteriousness predictions and binding affinity calculations. The conceptual framework presented here can be adapted to study other hormone precursor genes, and we hope to stimulate future studies involving *in vitro* characterizations of variants to examine the effect on ligand binding and signal transduction to expand our current knowledge on the mutational impact of receptor-peptide hormone interactions.

## Materials and Methods

### Dataset Generation and Integration

Throughout, we have defined *GCG* to be located to the region 2:162,142,882-162,152,404 in GRCh38 coordinates and 2:162,999,392-163,008,914 in GRCh37. We have used ENST00000418842.7 as canonical transcript and P01275 (GLUC_HUMAN) as UniProt identifier. UK Biobank variants, 200,629 exomes reflective of the general British population, were sourced from Data-Field 23156 version Oct 2020 and *GCG* loci were filtered with PLINK 2.0 (98). Variants were sourced from gnomAD v2.1.1 (non-TOPMed) (12), which consists of 122,439 exomes and 13,304 genomes from a variety of populations with a small fraction of samples known to have participated in either cancer or neurological studies. This yielded 114 missense variants which were subsequently remapped from GRCh37 to GRCH38 using the NCBI Genome Remapping Service (https://www.ncbi.nlm.nih.gov/genome/tools/remap). Variants were sourced from the TOPMed Freeze 8 on GRCh38 on the Bravo server (https://bravo.sph.umich.edu/freeze8/hg38/), containing 132,345 whole genomes. TOPMed aggregates >80 studies of various disease risk factors and prevalent diseases including heart and lung diseases. For variants present in more than one dataset, we let the allele frequency be the max allele frequency among the datasets. A variant was deemed a singleton if it was only observed once when looking at the datasets individually.

Peptide start/end positions were mapped from UniProt molecule processing information to positions in the Ensembl canonical transcript. To calculate the number of theoretically possible variants, we looped over the entire CDS as a Biopython mutableSeq object (99), substituting all possible bases at all position and translating the resulting codon to amino acid. If the substitution was non-synonymous, did not result in a stop-codon, and was unique, it was used to create a list of unique variants totaling 1075 variants.

For the UK Biobank individual data, samples were pulled as pVCF as described above and loaded into Hail (100) as a MatrixTable which was subsequently row-annotated with CADD and underlying annotations (see below) before being filtered for appropriate loci and consequence equalling “NON_SYNONYMOUS” excluding “start_lost”. Hail’s sample_qc and variant_qc functions were used to generate homo- and heterozygous counts.

### Calculations of Predicted Deleteriousness

Combined Annotation Dependent Depletion (CADD) scores were obtained by uploading the all 184 as a VCF file to the CADD web-server (https://cadd.gs.washington.edu/, release 1.5 (48)). Throughout the CADD PHRED score, normalized to all ~9 billion variants across the genome, was used. For primateAI, exome-wide pre-computed scores were downloaded from: https://github.com/Illumina/PrimateAI. Both sets of scores were added by indexing by position, ref, and alt allele.

### Conservation Scoring

We sourced *GCG* orthologue alignments from the All Species Set from Ensembl Compara release 103 (58). High Confidence orthologues were defined as having as having a minimum Gene Order Conservation (GOC) Score of 50, a minimum Whole Genome Alignment (WGA) Coverage of 50, a minimum % identity of 25 as suggested by Ensembl Compara (https://www.ensembl.org/info/genome/compara/Ortholog_qc_manual.html). Filtering for high confidence resulted in 222 high confidence orthologues from 164 vertebrates. Conservation scores, Rate4Site (46), were calculated for each position on the ConSurf server (https://consurf.tau.ac.il/) (47), using an empirical Bayesian method (101). Scores are normalized to 0 mean and 1 standard deviation. The most conserved position has a score of –1. Mean trace is Gaussian smoothed (scipy.ndimage.gaussian_filter1d with sigma=1), and 50% confidence interval is shown. Logo plots were generated from the abovementioned orthologue set using WebLogo (http://weblogo.threeplusone.com/) (57).

### Calculation of Stability Effects of Missense Mutations

We assessed the estimated stability effect of all human genetic missense variants using FoldX5.0 (56). FoldX employs energy terms weighted by empirical data from protein engineering experiments to provide a quantitative estimation of each mutant to the receptor-ligand complex stability. The energies for the WT (ΔG_fold,wt_) and mutant (ΔG_fold,mut_) receptor-ligand complex were computed to give the stability change ΔΔG_fold_ (kcal/mol) = ΔG_fold,mut_ − ΔG_fold,wt_ (61). We started by obtaining refined complex models for GLP-1/GLP1R (PDBid: 6X18) and glucagon/GCGR (PDBid: 6LMK) from GPCRdb, which pre-deposits refined experimental structures including repaired distorted regions, reverted mutated amino acids, and filled-in missing residues (102). To perform the stability analyses each complex was energy minimized with the FoldX ‘repair pdb’ function at 298K, pH 7.0, and 0.05M ion strength to optimize the structures by removing any steric clashes. To map the energetic landscape of each peptide complex, we mutated each residue to alanine and the respective missense variant using the command ‘BuildModel’. We calculated the average energy contribution and standard deviation for each genetic variant and alanine substitution after performing 10 independent runs to ensure the identification of the minimum energy conformations also for large residues, which possess many rotamers. We classified variants into categories based on the calculated energy difference in kcal/mol: highly destabilizing (ΔΔG > +1.84 kcal/mol), destabilizing (+0.92 to +1.84 kcal/mol), slightly destabilizing (+0.46 to +0.92 kcal/mol), neutral (-0.46 to –0.46 kcal/mol), slightly stabilizing (-0.92 to -0.46 kcal/mol), and stabilizing (-0.92 to -1.84 kcal/mol) (61).

## Data Availability Statement

The original contributions presented in the study are included in the article/**Supplementary Material**. Further inquiries can be directed to the corresponding authors.

## Author Contributions

Conceptualization: AH and MR. Methodology: AH and JM. Validation: JM and PL. Formal Analysis: JM and PL. Investigation: JM and PL. Resources: AH, JM, and PL. Data Curation: JM and PL. Writing – Original Draft: AH and PL. Writing – Review & Editing: AH, JM, PL, HB-O, and MR. Visualization: AH, JM, and PL. Supervision: AH, HB-O, and MR. Project Administration: AH and M.R. Funding Acquisition: HB-O, AH, and MR. All authors contributed to the article and approved the submitted version.

## Funding

We would like to gratefully acknowledge funding from the Lundbeck Foundation (R278-2018-180).

## Conflict of Interest

The authors declare that the research was conducted in the absence of any commercial or financial relationships that could be construed as a potential conflict of interest.
